# Relationship between clinical characteristics and survival of gastroenteropancreatic neuroendocrine neoplasms: A single-institution analysis (1995–2012) in South China

**DOI:** 10.1186/1472-6823-12-30

**Published:** 2012-11-29

**Authors:** Yu-hong Wang, Yuan Lin, Ling Xue, Jin-hui Wang, Min-hu Chen, Jie Chen

**Affiliations:** 1 Department of Gastroenterology, The First Affiliated Hospital of Sun Yat-sen University, 58 Zhongshan II Road, Guangzhou, People’s Republic of China; 2 Department of Pathology, The First Affiliated Hospital of Sun Yat-sen University, 58 Zhongshan II Road, Guangzhou, People’s Republic of China

**Keywords:** Gastroenteropancreatic neuroendocrine neoplasms, Clinical pathological characteristics, Survival

## Abstract

**Background:**

Gastroenteropancreatic neuroendocrine neoplasm (GEP-NEN) is the most common type of neuroendocrine tumors accounting for 65–75% of neuroendocrine neoplasms (NENs). Given the fact that there are few studies on GEP-NENs among Chinese patients, we performed a retrospective study in South China.

**Methods:**

Totally 178 patients with GEP-NENs treated at the First Affiliated Hospital of Sun Yat-sen University between January 1995 and May 2012 were analyzed retrospectively.

**Results:**

Pancreas was found the most common site of involvement (34.8%). 149 patients (83.7%) presented as non-functional tumors with non-specific symptoms such as abdominal pain (33.7%); carcinoid syndrome was not found in this study. Several methods are useful for localization of GEP-NENs, yielding varied detection rates from 77.8% to 98.7%. Positive rates of chromogranin A (CgA) and synaptophysin (Syn) immunhistochemically were 69.1% and 90.2%, respectively. 87 patients (51.5%) had G1 tumors, 31(18.3%) G2 tumors and 51 (30.2%) G3 tumors. Neuroendocrine tumor (NET), neuroendocrine carcinoma (NEC) and mixed adenoendocrine carcinoma (MANEC) were 69.8%, 27.2% and 3.0%, respectively. 28.1% of patients presented with distant disease. Surgery was performed in 152 (85.4%) patients, and overall 5-year survival rate was 54.5%. Functionality, G1 grading and NET classification were associated with favorable prognosis in univariate analysis. Distant metastasis contributed to unfavorable prognosis of these tumors.

**Conclusions:**

Nonfunctional tumors with non-specific symptoms account for the majority of GEP-NENs. Diagnosis depends on pathological classification. Multidisciplinary treatments could help improve the outcome.

## Background

Neuroendocrine neoplasms, which originate from neuroendocrine cells distributed throughout the body, comprise a heterogeneous family with a wide and complex clinical behaviors [1]. The incidence of NENs ranges from 2.5 to 5 cases per 100,000 in the United States, and the gastrointestinal tract is the most commonly affected site [2,3]. According to an analysis of the National Cancer Institute’s Surveillance, Epidemiology and End Results database (SEER, http://seer.cancer.gov/data/index.html), which is the largest epidemiologic series nowadays, the incidence of NENs has been rising substantially in the past 30 years.

NENs have been the subject of debate regarding optimal nomenclature, grading, staging and classification of these tumors for many years. A uniform World Health Organization (WHO) classification greatly facilitates the comparison of clinical, pathological and prognostic features and results of treatment in GEP-NENs, and so do the China Consensus Guidelines for the standards of histopathologic diagnosis as well [4,5].

The incidence of NENs, the treatments and survival of Caucasians have been well studied in western countries such as United States, Norway, Spain, German and the United Kingdom [2,3,6-9]. But for Asian population [10-13], especially for Chinese population, available information on these cancers is rather limited [14]. Therefore, it requires detailed data for comprehensive knowledge of NENs in China. Based on the 17-year data of our hospital, a comprehensive retrospective study was performed to examine the relationship between clinical pathological characteristic and survival of GEP-NENs. To our knowledge, it is the first study providing information on these tumors using the latest histopathologic diagnosis consensus from an Asian country.

## Methods

178 patients with histologically confirmed sporadic GEP-NENs from The First Affiliated Hospital, Sun Yat-sen University (1995–2012) were enrolled in this study to collect clinical information including age, gender, locations, clinical syndromes, endoscopic and radiographic features, histopathological characteristics, metastasis patterns, treatment modalities and outcomes.

The histology of each patient was reviewed according to the WHO classification [4] and China Consensus Guidelines [5]: First, immunohistochemical staining of CgA and Syn, which are all neuroendocrine markers, were performed to recognize the histological patterns of these tumors. Specific peptide hormones (eg. insulin, glucagon and somatostatin) staining methods were not regularly used only when a functional neuroendocrine neoplasm was considered. Second, the Ki-67 index (≤2%, 3–20%, and >20% per 500–2000 tumor cells in the most active regions or hot spots, respectively) or mitotic rate (1, 2–20, and >20 mitoses per 10 high-power field in the most active regions or hot spots, respectively), which was re-stained or recounted, was used to estimate the tumor proliferative activities. Tumors with a Ki-67 index of <2% were classified as G1 tumors, index of 3–20% were classified as G2, greater than 20% as G3. Likewise, tumors with mitotic rates of <2/10 HPF were classified as G1, those of 2 to 20/10 HPF were classified as G2, greater than 20/10 HPF as G3. Once the grading of Ki-67 index disaccorded with the mitotic rate, the higher one was preferred. Thus, GEP-NENs were classified as NET (G1 and G2), NEC (G3) and MANEC (G3).

Overall survival was defined as the time from diagnosis to death or last follow-up in living patients. Survival rate was estimated according to the Kaplan–Meier product limit method, and differences between subgroups were assessed by the log-rank test with P < 0.05 as statistically significant. SPSS 16.0 was used for statistical analysis.

The study was approved by the ethics committee of The First Affiliated Hospital Sun Yat-sen University (with a reference number: [2012]317) and complied with the Declaration of Helsinki.

## Results

### Clinical features

Among the 178 Chinese patients with GEP-NENs, 108 (60.7%) were men and 70 (39.3%) were women; male-to-female ratio was 1.54. The mean age was 50.96 ± 15.01 years. The most common sites were the pancreas (62/178, 34.8%), followed by rectum (36/178, 20.2%), stomach (25/178, 14.0%), duodenum (13/178, 7.3%), metastatic NENs of unknown primary (12/178, 6.7%) and esophagus (7/178, 3.9%). Other sites included appendix, jejunum/ileum, Vater’s ampulla at 12.9% (23/178). Non-functional tumors comprised the majority of GEP-NENs (149/178, 83.7%), whereas functional tumors accounted for the other 16.3%. A variety of gastrointestinal manifestations were caused by the effect of local compression on nearby tissues in nonfunctional tumors. The most common initial presentation was abdominal pain (60/178, 33.7%), which was not specific for the diagnosis of tumor. Other non-specific symptoms were gastrointestinal bleeding (29/178, 16.3%), jaundice (16/178, 9.0%), progressive dysphagia (9/178, 5.1%), diarrhea (8/178, 4.5%), abdominal distension (6/178, 3.4%) and so on. Incidental diagnosis occurred in 10.1% of cases which were usually asymptomatic. Insulinoma comprised 93.1% of functional tumors, which mainly occurred in pancreas, occasionally followed by the substantially rarer glucagonoma and vasoactive intestinal peptidoma (only 1 case respectively in our study). Typical symptoms included hypoglycemia, epileptic seizure and secondary diabetes mellitus, which heralded functional NENs, but carcinoid syndrome did not present in our study. The demographics and presenting symptoms of GEP-NENs are listed in Table 1.

**Table 1:** Characteristics of study population (N = 178 patients)

**Site**	**All patients**		**Men, n(%)**	**Women, n(%)**	**Clinical symptoms**	**Main signs**
	**N**	**%**				
Pancreas	62	34.8	32(51.6)	30(48.4)	Abdominal pain, Jaundice, Hypoglycaemia	Jaundice
Rectum	36	20.2	29(80.6)	7(19.4)	Gastrointestinal bleeding, Abdominal pain, Diarrhea	Rectum mass
Somach	25	14.0	16(64.0)	9(36.0)	Abdominal pain, Gastrointestinal bleeding, Dysphagia	Abdominal tenderness
Duodenum	13	7.3	10(76.9)	3(23.1)	Abdominal pain, Jaundice, Gastrointestinal bleeding	Jaundice
Metastasis of unknown primary	12	6.7	6(50.0)	6(50.0)	Abdominal pain, Asymptomatic, Fatigue	Hepatomegaly
Esophagus	7	3.9	5(71.4)	2(28.6)	Progressive dysphagia	No signs
Appendix	6	3.4	2(33.3)	4(66.7)	Abdominal pain, Abdominal distension	Rebound pain in the Mcburney’s point
Jejunum/ileum	4	2.2	3(75.0)	1(25.0)	Gastrointestinal bleeding, Small bowel obstruction	Anemia
Gallbladder	4	2.2	1(25.0)	3(75.0)	Jaundice, Asymptomatic	Jaundice
Vater’s ampulla	3	1.7	3(100)	0(0)	Jaundice, Abdominal pain,	Jaundice
Peritoneum	3	1.7	0(0)	3(100)	Abdominal pain, Asymptomatic	Abdominal mass
Cecum	1	0.6	1(100)	0(0)	Abdominal pain	Abdominal mass
Choledoch	1	0.6	1(100)	0(0)	Jaundice	Jaundice
Greater omentum	1	0.6	0(0)	1(100)	Asymptomatic	No signs

### Imaging studies

The most frequently used examination procedures included endoscopy, ultrasound, endoscopic ultrasonography (EUS), computed tomography (CT) scan, magnatic resonance imaging (MRI), and positron emission computed tomography imaging (PET-CT, using with 16 F-FDG). Endoscopy provided the highest detection rate of 98.7% (74/75). EUS was performed on 37 patients, of which a lesion was found in 34 patients, promised a detection rate of 91.9%. MRI and PET-CT, was performed in only about 10% of patients, respectively. Tumors usually appeared as polypoid prominences, ulcer type or cauliflower-like neoplasm under endoscopy; whereas on CT scan, they appeared as local space-occupying lesions which were significantly enhanced by iodinated contrast. Ultrasound and EUS usually demonstrated the tumors as rounded, homogeneous, hypoechoic, well-defined and well-vascularized masses (Table 2).

**Table 2:** Characteristics of imaging studies

**Imaging studies**	**Site**	**Manifestation**	**Case tested**	**Positive tests**	
				**n**	**%**
Endoscopy	gastrointestinal tract		75	74	98.7
Gastroscope	esophagus, stomach, duodenum	ulcer type, bulge type, invasive type	34	34	100
Duodenoscope	duodenum	bulge type	3	3	100
Small intestinal endoscope	jejunum/ileum	small intestinal hemorrhage	2	1	50.0
Colonoscope	rectum, appendix	polypoid prominences, submucosal uplift, cauliflower-like neoplasm	36	36	100
Ultrasound	pancreas, liver, gallbladder, cho- ledoch	hypoechoic masses, well delimited and vascularized	63	49	77.8
EUS	pancreas, duodenum, stomach	hypoechoic masses	37	34	91.9
CT scan	pancreas, liver, stomach	local space-occupying lesions	123	98	91.9
MRI	pancreas, duodenum, biliary	local space-occupying lesions	20	19	95.0
PET-CT	pancreas, rectum	local space-occupying lesions	20	19	95.0

EUS, endoscopic ultrasonography; CT, computed tomography; MRI, magnatic resonance imaging; PET-CT, positron emission computed tomography imaging.

### Pathologic characteristics

Overall, the mean diameter of tumors was 3.95 cm (0.4–25 cm): 38.6% were smaller than 2 cm in diameter, 29.7% ranging from 2 to 4 cm, and 31.7% larger than 4 cm. Immunohistochemistry staining determined a 69.1% positive rate of CgA and a 90.2% positive rate of Syn. Ki-67 index and mitotic rate were assessed in 127 and 118 specimens to estimate the proliferative activities. Over half (51.5%) of the tumors were G1, 18.3% were at G2 and 30.2% at G3. The most common tumor type was NET (69.8%), followed by NEC (27.2%) and MANEC (3.0%). Approximately half of the assessed tumors (53/100, 53.0%) originated from gastrointestinal tract and biliary system with muscularis or serosa infiltration at diagnosis. Local infiltration and lymphatic metastasis occurred in 23.0% and 27.0% of patients respectively. Distant metastasis was a frequent event at diagnosis with an occurrence of 23.0% (41/178), which increased to 28.1% (55/178) during follow up. The liver was one of most frequently involved organs: liver metastasis occurred in 44 (80.0%) of 55 patients in the disease courses. Among the 44 patients, 29 presented with synchronous liver metastasis, whereas other 15 presented with metachronous liver metastasis during follow-up. Other locations that tumors involved were the peritoneum (12.7%, 7/55), cavitas pelvis (9.1%, 5/55), bone (7.3%, 4/55) and ovary (5.5%, 3/55). The most common site of primary tumor associated with widespread disease at diagnosis was cecum (100.0%), followed by jejunum/ileum (75.0%), gallbladder (50.0%), duodenum (38.5%), Vater’s ampulla (33.3%) and stomach (28.0%) (Table 3).

**Table 3:** Pathologic characteristics

**Characteristics**	**Case tested**	**Positive tests**	
		**n**	**%**
Immunohistochemistry			
CgA	149	103	69.1
Syn	143	129	90.2
Tumor grading			
G1	169	87	51.5
G2	169	31	18.3
G3	169	51	30.2
Tumor type			
NET	169	118	69.8
NEC	169	46	27.2
MANEC	169	5	3.0
Infiltration/Metastasis			
Muscularis/Serosa infiltration	100	53	53.0
Adjacent tissue/Capsule infiltration	178	41	23.0
Lymphatic metastasis	178	48	27.0
Distant metastasis			
At initial diagnosis	178	41	23.0
During follow-up	178	55	28.1

CgA, Chromogranin A; Syn, Synaptophysin;NET, neuroendocrine tumor; NEC, neuroendocrine carcinoma; MANEC, mixed adenoendocrine carcinoma.

### Therapeutic interventions

85.4% patients underwent a surgery with curative intent (75.9%) or for palliative purpose (9.6%). Different types of endoscopic radical surgery were performed, including endoscopic mucosa resection (EMR), endoscopic submucosal dissection (ESD) and endoscopic electroexcision. Local-regional therapies such as transcatheter hepatic arterial chemoembolization (TACE), radiofrequency or other ablative techniques were carried out only in 11 cases (6.2% of the population). Chemotherapy and biological therapy were performed in 31 patients, among which 15 received chemo regimen, 8 received biological therapy and 8 received both. The most common first-line chemo combinations included platinum-etoposide (6 patients, 3.4%), oxaliplatin- capecitabine (2 patients, 1.3%), oxaliplatin-TS-1 (2 patients, 1.3%) and so on. Octreotide, a somatostatin analogue, was frequently administered at a dose of 20–40 mg/month as a biological therapy, combined with chemotherapy in 1 patient (0.6%) after surgery and in 7 patients (3.9%) with unresectable tumors. 14 (7.9%) cases with progressive malignant disease were treated only with supportive care.

### Survival and prognostic factors

**Figure 1:**
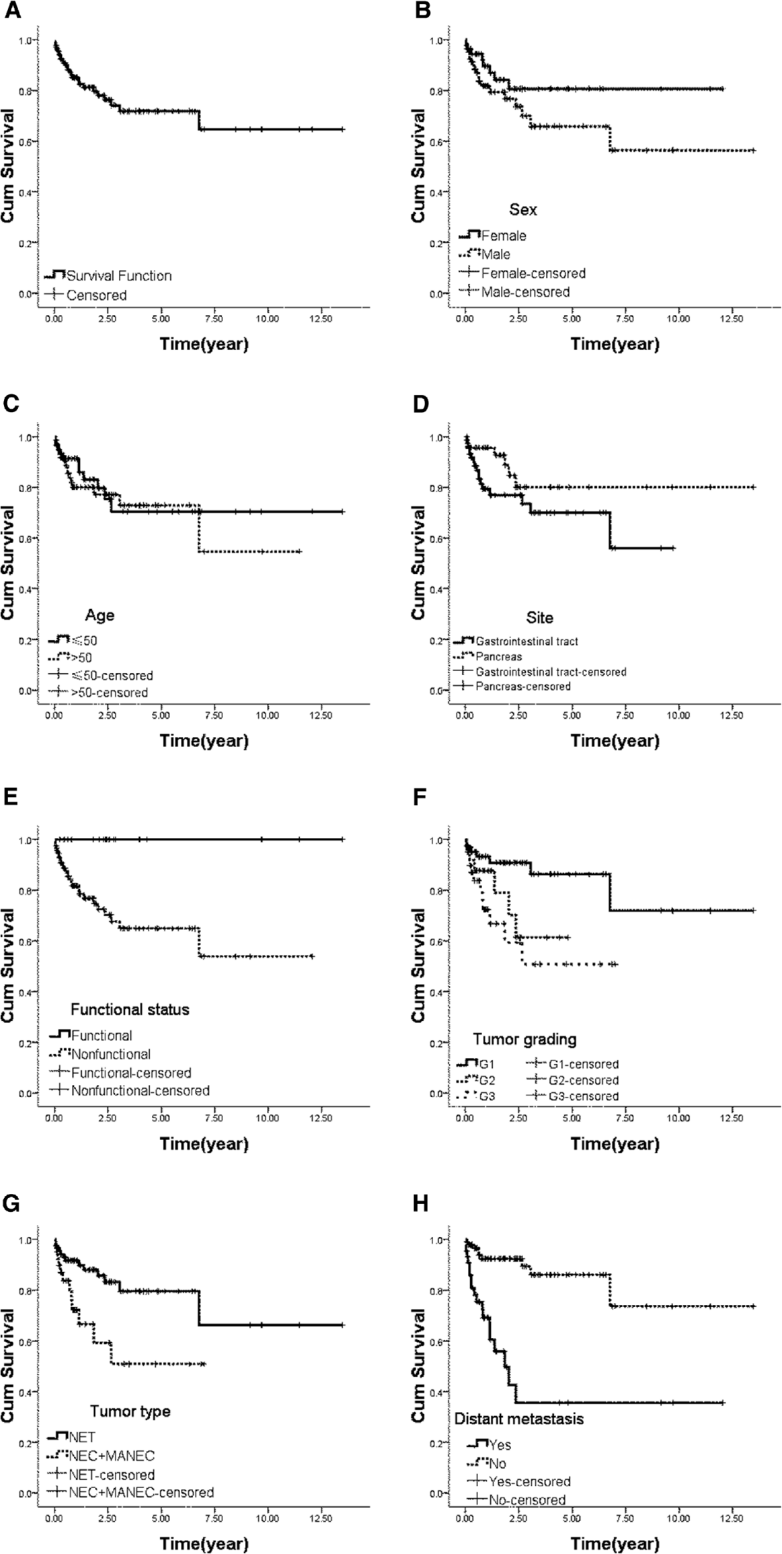
**Overall survival (A) Overall survival in all patients.** (**B**) Overall survival by sex. (**C**) Overall survival by age at diagnosis. (**D**) Overall survival by site of tumors. (**E**) Overall survival by functional status. (**F**) Overall survival by histological grading. (**G**) Overall survival by tumor type. (**H**) Overall survival by condition of distant metastasis.

136 out of 178 patients received long-term follow up with a median duration of 8.6 years (range 0.03–13.48 years). Median survival was not obtained during the observation period. The 1-, 3- and 5-year survival rates was 74.4%, 66.7% and 54.5% respectively, and 25 patients had died at the last follow-up (14.0%). The major causes of death were tumor-related complications (84.0%), and treatment-related adverse events (12.0%); other disease contributed the other 4.0%. An analysis was performed on patients’ age, gender, primary tumor site, histopathological grading, classification and condition of metastasis to identify prognostic factors for survival. Univariate analysis confirmed that functional tumors, patients were at G1 phase and classified as NET were superior to other types of NENs in survival. Distant metastasis also contributed to the prognosis of these neuroendocrine tumors. However, age, sex, primary tumor site had little impact on overall survival. The mean survival time and statistic data were provided in Table 4. Survival curves were displayed in Figure 1.

**Table 4:** Overall survival

**Factors**	**Overall survival**				
	**Number**	**Mean (years)**	**95% CI**	***χ*2**	**P**
All patients	136	9.5	8.1-11.0		
Sex				2.053	0.152
Female	54	9.9	8.5-11.3		
Male	82	8.7	6.7-10.6		
Age				0.259	0.611
≤50	60	9.9	8.0-11.8		
>50	76	7.8	5.9-9.6		
Site				2.385	0.123
Gastrointestinal tract	77	6.7	5.4-8.0		
Pancreas	46	11.1	9.3-12.9		
Functional status				6.691	0.006
Functional	22	NR	NC		
Nonfunctional	114	7.7	6.1-9.2		
Tumor grading				9.087	0.011
G1	63	10.8	8.7-12.9		
G2	25	3.5	2.6-4.3		
G3	41	4.1	2.8-5.4		
Tumor type				6.634	0.010
NET	88	10.1	8.1-12.1		
NEC + MANEC	41	4.1	2.8-5.4		
Distant metastasis				23.773	0.000
Yes	44	5.0	2.7-7.3		
No	92	11.0	9.2-12.8		

CI, confidence interval; NR, not reached; NC, not computable; NET, neuroendocrine tumor; NEC, neuroendocrine carcinoma; MANEC, mixed adenoendocrine carcinoma.

## Discussion

The WHO classification system of gastroenteropancreatic neuroendocrine tumors was adopted in previous studies [3,7,8,10-12,14]. Some of these studies only focused on particular types of GEP-NENs such as well-differentiated endocrine tumors, poorly differentiated endocrine carcinomas or a single site of tumors (pancreas, colon or rectum). Our study investigated the pathologic features of GEP-NENs by using the latest histopathologic diagnosis consensus for the first time. It also analyzed any possible tumor site of digestive system including pancreas, biliary and peritoneal cavity. This study should contribute to establishing a database of the epidemiology, clinical pathological features, treatment and prognosis of GEP-NENs in China.

It is confirmed in our study that GEP-NENs comprise a heterogeneous group in relation to their primary locations. Previous researches indicated that the small intestine and appendix were the most predominant NENs locations [2,15-17]. But according to our study, pancreas is the principal site of GEP-NENs. The rectum is the most frequent sites of gastrointestinal tract, followed by the stomach and duodenum, whereas the jejunum/ileum accounts for no more than 2% tumor cases. A similar distribution of NENs was also found from a Korean study [10], which observed that rectum was the most common primary site of tumor in 470 available cases, followed by the pancreas, stomach and duodenum. Results from another three registries including SEER, National Cancer Registry for Gastroenteropancreatic Neuroendocrine Tumors (RGETNE, http://www.retegep.net) and Norwegian Registry of Cancer (NRC) significantly differed from that in our series: Rectum and jejunum/ileum were the most common sites for NENs in the SEER Program tumor registry, pancreas NENs were only the third most common NENs; The pancreas and jejunum/ileum were the most frequent positions in RGETNE; whereas the small intestine was the most frequent sites of origin, followed by the colon and rectum in NRC. These inconsistencies may be due to the racial disparities, as well as the selection bias among population based data and hospital series. So a larger patient population is required to carry on further investigation.

NENs can be classified into functional and nonfunctional tumors according to the presence or absence of symptoms associated with hormones overproduction [18]. The current study demonstrated that the majority of nonfunctional NENs usually presented with non-specific symptoms, which may give rise to misdiagnosis of the tumors as irritable bowel syndrome or digestive adenocarcinomas. Our study also showed that insulinomas were the most frequently encountered functional tumors in the pancreas, accounting for 93.1% of pancreatic NENs. No case, however, presented with carcinoid syndrome in this study. Interestingly, the incidence of carcinoid syndrome (10–32%) in the Western population [8,17,19-21] is significantly different from our report, with the fact that ileal tumors account for the vast majority.

Assessments of the locations and extents of GEP-NENs were crucial for management. The present study analyzed imaging methods, which is commonly used in current clinical practice, in this patient population. Conventional imaging procedures include endoscopy, ultrasound, EUS, CT scan, MRI and PET-CT, with detection rates ranging from 77.8 to 98.7%. CT scan was one of the most widely used imaging modalities (123/178) whereas endoscopy promised the highest yields of tumor detection (98.7%). The introduction of EUS provides unique advantages in evaluating the pancreatic biliary system, especially in tumors <1.0 cm in diameter and micrometastasis. The typical EUS patterns of NENs includes rounded, homogeneous, hypoechoic, well defined and vascularized masses, with the detection rate of 91.9% in our study, rather comparable to the results achieved in other series [22-24]. Small tumors and liver metastasis (i.e., tumors <0.5 cm in diameter) may be missed, resulting in underestimate of the exact disease extent. No single technique is 100% sensitive and accurate. Therefore, multiple imaging modalities should be combined to detect small, biochemically diagnosed tumors.

Despite the advances in both morphology and biology, the classification of NENs is still under debate. The lack of a uniform classification system for NENs hampers evaluation of therapy and comparison between clinical trials [25]. European Neuroendocrine Tumor Society (ENETS) and the North American Neuroendocrine Tumor Society (NANETS) have published diagnosis standard and pathology reports of NENs in 2009 and 2010 [18,26], respectively. Furthermore, the WHO revised the nomenclature and classification of GEP-NENs in 2010, version 4 [4]. In 2011, China established her own classification system for NENs [5]. Chinese Pathologic Consensus Group suggested the term “Neuroendocrine neoplasm (NEN)” instead of “Neuroendocrine Tumor (NET)” and formulated the classification criteria by the use of Ki-67 index/mitotic rate and histology. The pathologic features of NENs in our hospital were reviewed according to this diagnosis consensus in the current analysis, which to our knowledge, is the first study using the newest consensus. Overall, G1 tumors accounted for 51.5% of 169 available cases, followed by G3 (30.2%) and G2 (18.3%). The occurence of NET, NEC and MANEC were 69.8%, 27.2% and 3.0%, respectively. The availability of this uniform system for NETs greatly facilitates classification of the tumors, evaluation of treatment, and comparison of clinical trials.

In our series, distant disease at initial diagnosis occurred at the rate of 23.0%, which increased to 28.1% during follow up. Liver was the most frequent site tumor involved and the distribution of distant metastasis was wider than that either in SEER or in NRC (18–22%). In RGETNE, however, a significant proportion of patients (44%) with widespread disease were reported compared with our series. The frequency of primary tumor sites associated with distant disease varied in different series: in our cohort, the most common sites was cecum (100.0%), followed by jejunum/ileum (75.0%), gallbladder (50.0%), duodenum (38.5%), Vater’s ampulla (33.3%) and stomach (28.0%); in the SEER Registry, the most common site was pancreas (64%), followed by cecum/colon (44%/32%) and jejunum/ileum (30%); and in the RGETNE Registry, it was jejunum/ileum (65%), followed by colon (48%) and rectum (40%). Therefore, jejunum/ileum tumors appear to have a greater propensity for distant metastasis. However, the diversity should be taken into account.

Among the many therapeutic options for NENs, surgery is the treatment of choice. A variety of operations are available to reduce load of tumor and improve survival. The extent of surgical resection depends on the tumor size and origin and approximately 75.9% of patients have undergone a radical surgery. Radiofrequency ablation or TACE is usually adopted to treat liver involvement, accounting for 6.2% of the cases.

Besides surgery, other therapeutic options such as chemotherapy, biological therapy and targeted therapy can be used for NENs. According to the new WHO 2010 classification, well-differentiated NENs are classified as G1 and G2 neuroendocrine tumors (NETs) and poor-differentiated NENs are referred to as G3 neuroendocrine carcinomas (NECs). It has been reported that existing cytotoxic chemotherapy agents have been of limited value for the treatment of well-differentiated gastrointestinal NENs (with response rates 10% ~ 15%) [27-29], but has been the standard of care for well-differentiated metastatic pancreatic endocrine tumors (with response rates 40% ~ 70%) [30-32]. However, chemotherapy is generally considered active in poor-differentiated NENs (with response rates 50% ~ 70%) [33-35]. According to the published documents, several chemotherapeutic regimens are available, most of them are either platinum based or flurouracil based [29,34,36,37]. For the GEP-NEC, platinum-based combination regimens with etoposide or paclitaxel [33,34,36] are recommended. In our cohort, chemotherapy was performed in 23 patients. The most frequently used chemo regimen was etoposide–platinum combination. During follow-up, 3 of them died of tumor progression. It has been noticed that biological therapy and targeted therapy promise some effect on NENs in recent years [38-43]. Somatostatin analogues are effective therapeutic option for functional neuroendocrine tumors because they reduce hormone-related symptoms [44-46]. They have also been shown to stabilize tumor growth over long periods, even to inhibit tumor growth in patients with well-differentiated metastatic neuroendocrine midgut tumors [40,47,48]. Although the treatment effect of somatostatin analogues on foregut and hindgut tumors remain to be confirmed, 16 patients including 2 patients with functional neuroendocrine tumors and 14 patients with well-differentiated metastatic GEP-NENs received long-term administration of octreotide LAR at a dose of 20–40 mg monthly in our study.

The prognosis of GEP-NENs is more favorable than that of the adenocarcinomas of the digestive system. The overall 5-year survival rate in our series was 54.5%, rather comparable to that of SEER or NRC registry [2,3,6] (50–59%), but it was lower than that in some European countries [7,9] (75–79%). The inconsistencies of survival rates may be due to the racial and geographical disparities. We also proved that prognosis differed statistically according to functional status, pathological grading and classification. As the great majority of functional tumors were insulinomas which are benign in most cases in our study, that may lead to the conclusion that functionality may be a favorable prognostic marker. The result obtained above may be caused by small sample in this series. We also confirmed that metastasis represented a worse outcome with a mean survival of 5.0 years (P = 0.000). Multivariate analysis was not done due to the small size of our series. Therefore, further evaluation in a larger patient population is required to estimate the independent prognostic factors of GEP-NENs.

A broad range of this heterogeneous tumors was reviewed in the current study, which to our knowledge, is the first report using the latest pathological diagnosis consensus of these tumors. We also confirmed that GEP-NENs may originate from any part of the digestive system, and the majority of them are nonfunctional tumors with non-specific symptoms. Endoscopy and radiographic examination play an important role in tumor detection. However, final diagnosis should be based on pathological detection. The prognosis of these tumors was more favorable compared with gastrointestinal carcinomas. Nonetheless, the outcome was extremely poor for patients with high grading tumor and distant metastasis. Further understanding of the molecular mechanisms should facilitate management of the disease. Early diagnosis is crucial for radical resection before development of local invasion or distant disease, and interdisciplinary cooperation is the direction of future.

## Conclusions

Nonfunctional tumors with non-specific symptoms account for the majority of GEP-NENs. Diagnosis depends on pathological classification. Multidisciplinary treatments could help improve the outcome.

## Competing interests

The authors declare that they have no competing interests.

## Authors’ contributions

WYH and LY contributed equally to this work; WYH and LY: Collection and/or assembly of data, Data analysis and interpretation, Manuscript writing; LY and XL: Pathological data collection and analysis; WJH: Collection of clinical data; Chen J and Chen MH: Conception and design, Financial and administrative support, manuscript editing. All authors read and approved the final manuscript.

## Pre-publication history

The pre-publication history for this paper can be accessed here:

http://www.biomedcentral.com/1472-6823/12/30/prepub

## References

[B1] Modlin IM Oberg K Chung DC Jensen RT de Herder WW Thakker RV Caplin M Delle Fave G Kaltsas GA Krenning EP Gastroenteropancreatic neuroendocrine tumours Lancet Oncol 2008 9 1 61 72 10.1016/S1470-2045(07)70410-2 18177818

[B2] Modlin IM Lye KD Kidd M A 5-decade analysis of 13,715 carcinoid tumors Cancer 2003 97 4 934 959 10.1002/cncr.11105 12569593

[B3] Yao JC Hassan M Phan A Dagohoy C Leary C Mares JE Abdalla EK Fleming JB Vauthey JN Rashid A One hundred years after “carcinoid”: epidemiology of and prognostic factors for neuroendocrine tumors in 35,825 cases in the United States J Clin Oncol 2008 26 18 3063 3072 10.1200/JCO.2007.15.4377 18565894

[B4] Bosman FT Carneiro F Hruban RH Theise ND WHO classification of tumours of the digestive system 2010 4 Lyon: International Agency for Research on Cancer

[B5] Chinese Pathologic Consensus Group for Gastrointestinal and Pancreatic Neuroendocrine Neoplasm China Consensus Guidelines for the standards of histopathologic diagnosis in Gastroenteropancreatic Neuroendocrine neoplasm Chin J Pathol 2011 40 4 257 262 21616002

[B6] Hauso O Gustafsson BI Kidd M Waldum HL Drozdov I Chan AK Modlin IM Neuroendocrine tumor epidemiology: contrasting Norway and North America Cancer 2008 113 10 2655 2664 10.1002/cncr.23883 18853416

[B7] Garcia-Carbonero R Capdevila J Crespo-Herrero G Diaz-Perez JA Martinez Del Prado MP Alonso Orduna V Sevilla-Garcia I Villabona-Artero C Beguiristain-Gomez A Llanos-Munoz M Incidence, patterns of care and prognostic factors for outcome of gastroenteropancreatic neuroendocrine tumors (GEP-NETs): results from the National Cancer Registry of Spain (RGETNE) Ann Oncol 2010 21 9 1794 1803 10.1093/annonc/mdq022 20139156

[B8] Ploeckinger U Kloeppel G Wiedenmann B Lohmann R The German NET-registry: an audit on the diagnosis and therapy of neuroendocrine tumors Neuroendocrinology 2009 90 4 349 363 10.1159/000242109 19776553

[B9] Lepage C Rachet B Coleman MP Survival from malignant digestive endocrine tumors in England and Wales: a population-based study Gastroenterology 2007 132 3 899 904 10.1053/j.gastro.2007.01.006 17383419

[B10] Lim T Lee J Kim JJ Lee JK Lee KT Kim YH Kim KW Kim S Sohn TS Choi DW Gastroenteropancreatic neuroendocrine tumors: incidence and treatment outcome in a single institution in Korea Asia Pac J Clin Oncol 2011 7 3 293 299 10.1111/j.1743-7563.2011.01423.x 21884442

[B11] Li AF Hsu CY Li A Tai LC Liang WY Li WY Tsay SH Chen JY A 35-year retrospective study of carcinoid tumors in Taiwan: differences in distribution with a high probability of associated second primary malignancies Cancer 2008 112 2 274 283 10.1002/cncr.23159 18008361

[B12] Konishi T Watanabe T Kishimoto J Kotake K Muto T Nagawa H Prognosis and risk factors of metastasis in colorectal carcinoids: results of a nationwide registry over 15 years Gut 2007 56 6 863 868 10.1136/gut.2006.109157 17213340 PMC1954860

[B13] Ito T Tanaka M Sasano H Osamura YR Sasaki I Kimura W Takano K Obara T Ishibashi M Nakao K Preliminary results of a Japanese nationwide survey of neuroendocrine gastrointestinal tumors J Gastroenterol 2007 42 6 497 500 10.1007/s00535-007-2056-6 17671766

[B14] Wang DS Zhang DS Qiu MZ Wang ZQ Luo HY Wang FH Li YH Xu RH Prognostic factors and survival in patients with neuroendocrine tumors of the pancreas Tumour Biol 2011 32 4 697 705 10.1007/s13277-011-0170-9 21479734

[B15] Maggard MA O’Connell JB Ko CY Updated population-based review of carcinoid tumors Ann Surg 2004 240 1 117 122 10.1097/01.sla.0000129342.67174.67 15213627 PMC1356383

[B16] Van Gompel JJ Sippel RS Warner TF Chen H Gastrointestinal carcinoid tumors: factors that predict outcome World J Surg 2004 28 4 387 392 10.1007/s00268-003-7019-3 14994141

[B17] Onaitis MW Kirshbom PM Hayward TZ Quayle FJ Feldman JM Seigler HF Tyler DS Gastrointestinal carcinoids: characterization by site of origin and hormone production Ann Surg 2000 232 4 549 556 10.1097/00000658-200010000-00010 10998653 PMC1421187

[B18] Klimstra DS Modlin IR Adsay NV Chetty R Deshpande V Gonen M Jensen RT Kidd M Kulke MH Lloyd RV Pathology reporting of neuroendocrine tumors: application of the Delphic consensus process to the development of a minimum pathology data set Am J Surg Pathol 2010 34 3 300 313 10.1097/PAS.0b013e3181ce1447 20118772

[B19] Pape UF Bohmig M Berndt U Tiling N Wiedenmann B Plockinger U Survival and clinical outcome of patients with neuroendocrine tumors of the gastroenteropancreatic tract in a german referral center Ann N Y Acad Sci 2004 1014 222 233 10.1196/annals.1294.025 15153439

[B20] Shebani KO Souba WW Finkelstein DM Stark PC Elgadi KM Tanabe KK Ott MJ Prognosis and survival in patients with gastrointestinal tract carcinoid tumors Ann Surg 1999 229 6 815 821 discussion 822–813. 10.1097/00000658-199906000-00008 10363895 PMC1420828

[B21] Vinik AI Thompson N Eckhauser F Moattari AR Clinical features of carcinoid syndrome and the use of somatostatin analogue in its management Acta Oncol 1989 28 3 389 402 10.3109/02841868909111212 2663049

[B22] Anderson MA Carpenter S Thompson NW Nostrant TT Elta GH Scheiman JM Endoscopic ultrasound is highly accurate and directs management in patients with neuroendocrine tumors of the pancreas Am J Gastroenterol 2000 95 9 2271 2277 10.1111/j.1572-0241.2000.02480.x 11007228

[B23] Zimmer T Scherubl H Faiss S Stolzel U Riecken EO Wiedenmann B Endoscopic ultrasonography of neuroendocrine tumours Digestion 2000 62 Suppl 1 45 50 10940687 10.1159/000051855

[B24] Varas Lorenzo MJ Miquel Collell JM Maluenda Colomer MD Boix Valverde J Armengol Miro JR Preoperative detection of gastrointestinal neuroendocrine tumors using endoscopic ultrasonography Rev Esp Enferm Dig 2006 98 11 828 883 17198475 10.4321/s1130-01082006001100004

[B25] Modlin IM Moss SF Chung DC Jensen RT Snyderwine E Priorities for improving the management of gastroenteropancreatic neuroendocrine tumors J Natl Cancer Inst 2008 100 18 1282 1289 10.1093/jnci/djn275 18780869 PMC2538549

[B26] Kloppel G Couvelard A Perren A Komminoth P McNicol AM Nilsson O Scarpa A Scoazec JY Wiedenmann B Papotti M ENETS Consensus Guidelines for the Standards of Care in Neuroendocrine Tumors: towards a standardized approach to the diagnosis of gastroenteropancreatic neuroendocrine tumors and their prognostic stratification Neuroendocrinology 2009 90 2 162 166 10.1159/000182196 19060454

[B27] Bukowski RM Tangen CM Peterson RF Taylor SA Rinehart JJ Eyre HJ Rivkin SE Fleming TR Macdonald JS Phase II trial of dimethyltriazenoimidazole carboxamide in patients with metastatic carcinoid. A Southwest Oncology Group study Cancer 1994 73 5 1505 1508 10.1002/1097-0142(19940301)73:5<1505::AID-CNCR2820730530>3.0.CO;2-V 8111718

[B28] Ansell SM Pitot HC Burch PA Kvols LK Mahoney MR Rubin J A Phase II study of high-dose paclitaxel in patients with advanced neuroendocrine tumors Cancer 2001 91 8 1543 1548 10.1002/1097-0142(20010415)91:8<1543::AID-CNCR1163>3.0.CO;2-N 11301403

[B29] Sun W Lipsitz S Catalano P Mailliard JA Haller DG Phase II/III study of doxorubicin with fluorouracil compared with streptozocin with fluorouracil or dacarbazine in the treatment of advanced carcinoid tumors: Eastern Cooperative Oncology Group Study E1281 J Clin Oncol 2005 23 22 4897 4904 10.1200/JCO.2005.03.616 16051944

[B30] Moertel CG Hanley JA Johnson LA Streptozocin alone compared with streptozocin plus fluorouracil in the treatment of advanced islet-cell carcinoma N Engl J Med 1980 303 21 1189 1194 10.1056/NEJM198011203032101 6252466

[B31] Moertel CG Lefkopoulo M Lipsitz S Hahn RG Klaassen D Streptozocin-doxorubicin, streptozocin-fluorouracil or chlorozotocin in the treatment of advanced islet-cell carcinoma N Engl J Med 1992 326 8 519 523 10.1056/NEJM199202203260804 1310159

[B32] Strosberg JR Fine RL Choi J Nasir A Coppola D Chen DT Helm J Kvols L First-line chemotherapy with capecitabine and temozolomide in patients with metastatic pancreatic endocrine carcinomas Cancer 2011 117 2 268 275 10.1002/cncr.25425 20824724 PMC4665634

[B33] Moertel CG Kvols LK O’Connell MJ Rubin J Treatment of neuroendocrine carcinomas with combined etoposide and cisplatin. Evidence of major therapeutic activity in the anaplastic variants of these neoplasms Cancer 1991 68 2 227 232 10.1002/1097-0142(19910715)68:2<227::AID-CNCR2820680202>3.0.CO;2-I 1712661

[B34] Hainsworth JD Spigel DR Litchy S Greco FA Phase II trial of paclitaxel, carboplatin, and etoposide in advanced poorly differentiated neuroendocrine carcinoma: a Minnie Pearl Cancer Research Network Study J Clin Oncol 2006 24 22 3548 3554 10.1200/JCO.2005.05.0575 16877720

[B35] Fjallskog ML Granberg DP Welin SL Eriksson C Oberg KE Janson ET Eriksson BK Treatment with cisplatin and etoposide in patients with neuroendocrine tumors Cancer 2001 92 5 1101 1107 10.1002/1097-0142(20010901)92:5<1101::AID-CNCR1426>3.0.CO;2-V 11571721

[B36] Mitry E Baudin E Ducreux M Sabourin JC Rufie P Aparicio T Lasser P Elias D Duvillard P Schlumberger M Treatment of poorly differentiated neuroendocrine tumours with etoposide and cisplatin Br J Cancer 1999 81 8 1351 1355 10.1038/sj.bjc.6690325 10604732 PMC2362979

[B37] Kouvaraki MA Ajani JA Hoff P Wolff R Evans DB Lozano R Yao JC Fluorouracil, doxorubicin, and streptozocin in the treatment of patients with locally advanced and metastatic pancreatic endocrine carcinomas J Clin Oncol 2004 22 23 4762 4771 10.1200/JCO.2004.04.024 15570077

[B38] Yao JC Phan AT Chang DZ Wolff RA Hess K Gupta S Jacobs C Mares JE Landgraf AN Rashid A Efficacy of RAD001 (everolimus) and octreotide LAR in advanced low- to intermediate-grade neuroendocrine tumors: results of a phase II study J Clin Oncol 2008 26 26 4311 4318 10.1200/JCO.2008.16.7858 18779618 PMC2653122

[B39] Kulke MH Lenz HJ Meropol NJ Posey J Ryan DP Picus J Bergsland E Stuart K Tye L Huang X Activity of sunitinib in patients with advanced neuroendocrine tumors J Clin Oncol 2008 26 20 3403 3410 10.1200/JCO.2007.15.9020 18612155

[B40] Rinke A Muller HH Schade-Brittinger C Klose KJ Barth P Wied M Mayer C Aminossadati B Pape UF Blaker M Placebo-controlled, double-blind, prospective, randomized study on the effect of octreotide LAR in the control of tumor growth in patients with metastatic neuroendocrine midgut tumors: a report from the PROMID Study Group J Clin Oncol 2009 27 28 4656 4663 10.1200/JCO.2009.22.8510 19704057

[B41] Yao JC Lombard-Bohas C Baudin E Kvols LK Rougier P Ruszniewski P Hoosen S St Peter J Haas T Lebwohl D Daily oral everolimus activity in patients with metastatic pancreatic neuroendocrine tumors after failure of cytotoxic chemotherapy: a phase II trial J Clin Oncol 2010 28 1 69 76 10.1200/JCO.2009.24.2669 19933912 PMC4295034

[B42] Yao JC Shah MH Ito T Bohas CL Wolin EM Van Cutsem E Hobday TJ Okusaka T Capdevila J de Vries EG Everolimus for advanced pancreatic neuroendocrine tumors N Engl J Med 2011 364 6 514 523 10.1056/NEJMoa1009290 21306238 PMC4208619

[B43] Raymond E Dahan L Raoul JL Bang YJ Borbath I Lombard-Bohas C Valle J Metrakos P Smith D Vinik A Sunitinib malate for the treatment of pancreatic neuroendocrine tumors N Engl J Med 2011 364 6 501 513 10.1056/NEJMoa1003825 21306237

[B44] Oberg K Kvols L Caplin M Delle Fave G de Herder W Rindi G Ruszniewski P Woltering EA Wiedenmann B Consensus report on the use of somatostatin analogs for the management of neuroendocrine tumors of the gastroenteropancreatic system Ann Oncol 2004 15 6 966 973 10.1093/annonc/mdh216 15151956

[B45] Karashima T Cai RZ Schally AV Effects of highly potent octapeptide analogs of somatostatin on growth hormone, insulin and glucagon release Life Sci 1987 41 8 1011 1019 10.1016/0024-3205(87)90690-4 2886886

[B46] Oberg K Future aspects of somatostatin-receptor-mediated therapy Neuroendocrinology 2004 80 Suppl 1 57 61 15477719 10.1159/000080743

[B47] Faiss S Rath U Mansmann U Caird D Clemens N Riecken EO Wiedenmann B Ultra-high-dose lanreotide treatment in patients with metastatic neuroendocrine gastroenteropancreatic tumors Digestion 1999 60 5 469 476 10.1159/000007693 10473972

[B48] Welin SV Janson ET Sundin A Stridsberg M Lavenius E Granberg D Skogseid B Oberg KE Eriksson BK High-dose treatment with a long-acting somatostatin analogue in patients with advanced midgut carcinoid tumours Eur J Endocrinol/European Federation of Endocrine Societies 2004 151 1 107 112 10.1530/eje.0.1510107 15248829

